# Efficacy of acupuncture for sleep disorders in amnestic mild cognitive impairment: a randomized controlled trial with fMRI

**DOI:** 10.3389/fneur.2026.1913478

**Published:** 2026-10-06

**Authors:** Di Qiu, Ruiyao Li, Jikang Yang, Liyao Ding, Jiayong Deng, Yongfeng Liu, Jinhuan Zhang

**Affiliations:** 1 Department of Acupuncture, The Fourth Clinical Medical College of Guangzhou University of Chinese Medicine, Shenzhen, China; 2 Department of Acupuncture, Shenzhen Traditional Chinese Medicine Anorectal Hospital, Shenzhen, China; 3 Department of Acupuncture, Shenzhen Traditional Chinese Medicine Hospital, Shenzhen, China

**Keywords:** acupuncture, amnestic mild cognitive impairment, functional magnetic resonance imaging, RCT - randomized controlled trial, sleep disorders

## Abstract

**Objective:**

To access the efficacy of acupuncture in improving sleep disorders in patients with amnestic Mild Cognitive Impairment (aMCI) and to investigate its mechanisms.

**Methods:**

Thirty aMCI patients with sleep disorders and fifteen healthy controls (HC) were enrolled. Patients were randomly assigned to verum acupuncture group (VA) or sham acupuncture group (SA) for a 12-week treatment. Assessments included the Montreal Cognitive Assessment (MoCA), Pittsburgh Sleep Quality Index (PSQI), and resting-state Functional Magnetic Resonance Imaging (fMRI).

**Results:**

At baseline, patients with aMCI exhibited aberrant brain activity in temporal, frontal, parietal, and cerebellar regions compared with HCs. After treatment, the VA group, but not the SA group, showed significant improvements in both MoCA and PSQI scores. Rs-fMRI analyses revealed significant VA-related changes in the amplitude of low-frequency fluctuation (ALFF) in the left calcarine sulcus and regional homogeneity (ReHo) in the cerebellar vermis. Changes in these imaging indicators were significantly correlated with changes in MoCA scores but not with changes in PSQI scores. Moreover, changes in MoCA scores were negatively correlated with changes in PSQI scores in the VA group (*r* = −0.536, *p* = 0.039).

**Conclusion:**

Acupuncture could effectively improve sleep and cognitive function in aMCI patients. The treatment-related alterations in brain function may be associated with changes in the dermatome layer and the brain-cerebellar cortex circuitry.

**Clinical trial registration:**

https://itmctr.ccebtcm.org.cn/mgt/search, Identifier ITMCTR2026000708.

## 1 Introduction

With global population aging, Alzheimer’s disease (AD) poses a major public health challenge. Cases are projected to rise from 55 million in 2019 to 139 million by 2050, creating a vast socioeconomic burden (1, 2). Amnestic mild cognitive impairment (aMCI), the prodromal stage of AD with an annual conversion rate of ~14.9%, is a critical intervention target (3).

Sleep disturbances are highly prevalent in aMCI (21–54%), significantly exceeding rates in healthy elders (4). A bidirectional, vicious cycle exists: chronic sleep disruption exacerbates AD pathology through multiple pathways, including impaired glymphatic clearance of β-amyloid (Aβ) (5), promoting Tau phosphorylation (6), neuroinflammation (7), dysregulation of neurotransmitter systems (8, 9), disruption of the circadian melatonin pathway (10, 11), and activation of specific signaling pathways that may exacerbate Aβ aggregation (12). Conversely, pre-existing brain network dysfunction in aMCI further disrupts sleep regulation, reinforcing the cycle (13). Traditional sedative-hypnotic medications carry side effects such as an increased risk of falls, drug dependence, and exacerbated cognitive impairment in the elderly (14, 15). Therefore, there is an urgent need to identify a non-pharmacological intervention method.

Therefore, this study employs resting-state fMRI to investigate the brain mechanisms of acupuncture for sleep disorders in aMCI, aiming to integrate traditional Chinese medicine theory with modern neuroimaging approaches.

Acupuncture, as a complementary therapy, has shown potential to improve both cognitive function and sleep outcomes (16, 17). Existing neuroimaging evidence provides preliminary insights into its central mechanisms. In patients with aMCI, acupuncture-related alterations have been identified in the dorsolateral prefrontal cortex and hippocampal functional connectivity, suggesting potential modulation of neural circuits involved in memory processing and cognitive control (18). Neuroimaging reviews and meta-analyses further suggest that acupuncture may modulate the hippocampus, anterior cingulate cortex, insula, thalamus, and prefrontal regions involved in memory processing, cognitive control, and large-scale network integration (19, 20). In patients with insomnia, acupuncture has been associated with changes in spontaneous activity within frontal and default mode network regions (21). A recent sham-controlled fMRI trial also demonstrated acupuncture-related modulation of functional connectivity among the anterior cingulate cortex, hippocampus, amygdala, thalamus, and dorsolateral prefrontal cortex, implicating neural systems involved in emotional processing and arousal regulation (22). Although these findings collectively implicate prefrontal, limbic, and large-scale brain networks in the effects of acupuncture, the reported neural alterations have varied across studies in terms of anatomical localization and neuroimaging measures, making it difficult to identify a consistent mechanistic pattern. More importantly, previous studies have generally investigated aMCI and insomnia separately and interpreted acupuncture-related changes within a single-diagnosis framework. It therefore remains unclear whether acupuncture can modulate the overlapping neural substrates of sleep and cognition and whether such neural changes are associated with concurrent improvements in both domains among patients with aMCI accompanied by sleep disturbance.

To address this knowledge gap, the present study investigated acupuncture-induced changes in spontaneous brain activity and examined their associations with improvements in sleep and cognitive function in this specific population.

## 2 Materials and methods

### 2.1 Participants

Between April 2024 and January 2025, 30 patients with aMCI and comorbid sleep disorders, as well as 15 HC, were recruited from the outpatient department of Shenzhen Hospital of Traditional Chinese Medicine and through online advertisements.

The inclusion criteria for sleep disorders in aMCI were as follows: (1) fulfillment of the diagnostic criteria for aMCI proposed by Petersen (23); (2) fulfillment of the diagnostic criteria for insomnia according to the Chinese Guidelines for the Diagnosis and Treatment of Insomnia (24); (3) age between 50 and 75 years; (4) a disease duration of more than 3 months accompanied by subjective memory complaints; (5) a MoCA score of 20–25, a Clinical Dementia Rating (CDR) score of 0.5, and a Pittsburgh Sleep Quality Index (PSQI) score greater than 5; and (6) preserved independence in activities of daily living.

The exclusion criteria were as follows: (1) receipt of acupuncture treatment or cognitive interventions within the previous month; (2) severe neurological disorders, major psychiatric illnesses, or other diseases known to affect cognitive function; (3) structural brain abnormalities detected on MRI or contraindications to MRI examination (e.g., claustrophobia or metallic implants); and (4) inability to complete clinical assessments or comply with study procedures.

Healthy controls were age-matched individuals (50–75 years) without subjective cognitive complaints or sleep disturbances, with normal neurological examinations, a MoCA score ≥26, and a PSQI score ≤ 5.

All participants provided written informed consent before enrollment. The study was approved by the Ethics Committee of Shenzhen Traditional Chinese Medicine Hospital (Approval No. K2025-020-01) and was conducted in accordance with the Declaration of Helsinki.

### 2.2 Sample size calculation

There is currently no precise method for estimating the sample size of acupuncture-related neuroimaging clinical trials. According to internationally recognized reviews, the sample size for each group should be at least 12 cases (25, 26). Considering data loss and exclusion from later data analysis, this trial will require 15 subjects per group, for a total of 30 cases.

### 2.3 Randomization and blinding

Thirty patients with aMCI were randomly assigned in a 1:1 ratio to either the verum acupuncture (VA) or the sham acupuncture (SA) using a computer-generated randomization sequence created with SPSS version 27.0, resulting in 15 participants per group. Due to the nature of the acupuncture intervention, a participant-, assessor-, and statistician-blinded design was employed. Participants, outcome assessors, and data analysts remained blinded to treatment allocation throughout the study. The randomization code was concealed and was not revealed until all clinical assessments, neuroimaging analyses, and statistical analyses had been completed. Randomization was performed by an independent researcher who was not involved in participant recruitment, treatment administration, outcome assessment, or data analysis.

### 2.4 Acupuncture treatment

The VA group protocol included the following acupoints: Baihui (GV20), Sishencong (EX-HN1), Shenting (GV24), Yintang (EX-HN3), Qihai (CV6), Guanyuan (CV4), Hegu (LI4), Shenmen (HT7), Zusanli (ST36), Fenglong (ST40), Sanyinjiao (SP6), Xuanzhong (GB39), Taixi (KI3), and Taichong (LR3).

Disposable sterile acupuncture needles (0.30 × 40 mm; Huanqiu, Suzhou Medical Appliance Factory, China) were used. Unless otherwise specified, needles were inserted perpendicularly to the skin. Baihui (GV20), Sishencong (EX-HN1), and Shenting (GV24) were inserted subcutaneously to a depth of 0.5–0.8 cun, with the needle tips at Sishencong directed toward Baihui, forming a convergent pattern. Yintang (EX-HN3) was inserted subcutaneously to a depth of 0.3–0.5 cun. Qihai (CV6), Guanyuan (CV4), Sanyinjiao (SP6), and Fenglong (ST40) were inserted perpendicularly to a depth of 1–1.5 cun. Shenmen (HT7) was needled to a depth of 0.3–0.5 cun; Hegu (LI4), 0.5–1 cun; Zusanli (ST36), 1–2 cun; and Xuanzhong (GB39), Taixi (KI3), and Taichong (LR3), 0.5–0.8 cun.

Each treatment session lasted 30 min. Acupuncture was administered three times weekly for 12 consecutive weeks, resulting in a total of 36 treatment sessions. Participants in the SA group received treatment using the Streitberger placebo needle method. All interventions were performed by the same licensed acupuncturist with more than 5 years of clinical experience. Manual stimulation was applied in the VA group to induce the deqi sensation, which was characterized by soreness, numbness, distension, or heaviness. After completion of the 12-week intervention, participants in the SA group were not provided with compensatory verum acupuncture.

### 2.5 Data collection

All patients underwent clinical assessments and MRI scanning before the first acupuncture session and again 72 h after the final session. HCs underwent the same assessments on a single occasion. Global cognitive function was evaluated using the Mini-Mental State Examination (MMSE) and Montreal Cognitive Assessment (MoCA), with higher scores indicating better cognitive performance. Episodic memory was assessed using the Chinese version of the Auditory Verbal Learning Test (AVLT), comprising three immediate recall trials (N1–N3), 5-min delayed recall (N4), 20-min delayed recall (N5), cued recall (N6), and recognition (N7). Higher AVLT scores indicated better memory performance.

For patients in the treatment groups, a post-treatment MRI scanning was acquired after completion of the entire course of acupuncture. All MRI data were collected using a Siemens 3.0 T scanner equipped with a 48-channel head coil. To minimize motion artifacts, participants were instructed to keep their eyes closed, remain still, and avoid systematic thinking during scanning.

The rs-fMRI parameters were as follows: field of view (FOV) = 256 × 256 mm^2^, repetition time (TR) = 2000 ms, echo time (TE) = 30 ms, 37 axial slices, matrix = 64 × 64, flip angle (FA) = 90°, voxel size = 3.75 × 3.75 × 4.0 mm^3^, and 240 volumes acquired. In addition, high-resolution three-dimensional T1-weighted anatomical images were obtained using a magnetization-prepared rapid gradient-echo (MPRAGE) sequence with the following settings: TR = 2,200 ms, TE = 2.45 ms, FOV = 256 × 256 mm^2^, 175 contiguous slices (no gap), and FA = 8°.

### 2.6 Data preprocessing

All research data were processed using the SPM12 toolkit on the MATLAB 2022a platform and the DPARSF module in DPABI (accessed at: http://rfmri.org/DPABI) (27). Detailed processing steps were as follows: (1) Conversion of image data from DICOM to NIFTI format; (2) Removal of the first 10 time points; (3) Slice timing correction; (4) Exclusion of data if head motion exceeded 2 mm translation or 2° rotation; (5) Image registration, aligning each subject’s T1 structural image with their BOLD image; (6) Skull stripping; (7) Spatial normalization using the DARTEL algorithm (28); (8) Nuisance covariate regression, including Friston’s 24 head motion parameters (29), global mean signal, white matter signal, and cerebrospinal fluid signal; (9) Application of a band-pass filter (0.01–0.10 Hz); and (10) Spatial smoothing with a Gaussian kernel of 6 mm full width at half maximum.

### 2.7 Statistical analysis

Neuroimaging analyses were conducted using the DPABI toolbox (version 5.4). Whole-brain comparisons of ALFF and ReHo maps were performed using two-sample *t*-tests, with age, sex, and years of education included as covariates. Multiple-comparison correction was performed using Gaussian Random Field (GRF) theory, with a voxel-level threshold of *p* < 0.05 and a cluster-level threshold of *p* < 0.05. Brain regions showing significant between-group differences were defined as regions of interest (ROI), and the corresponding ALFF and ReHo values were extracted for further analyses. Paired *t*-tests were conducted to evaluate within-group changes in ROI values before and after treatment. Pearson or Spearman correlation analyses, as appropriate, were performed to examine associations between neuroimaging metrics and clinical measures. Statistical significance was set at *p* < 0.05.

## 3 Results

### 3.1 Demographic and clinical characteristics

A total of 30 patients with aMCI and 15 HCs were included in the study (Figure 1). The 30 patients with aMCI were assigned to the VA and SA groups, with 15 participants in each group. The demographic and clinical characteristics of the participants are summarized in Table 1. No significant differences in age, sex, or years of education were observed among the HC, VA, and SA groups. Moreover, the VA and SA groups showed no significant differences in clinical outcomes at baseline.

**Figure 1:**
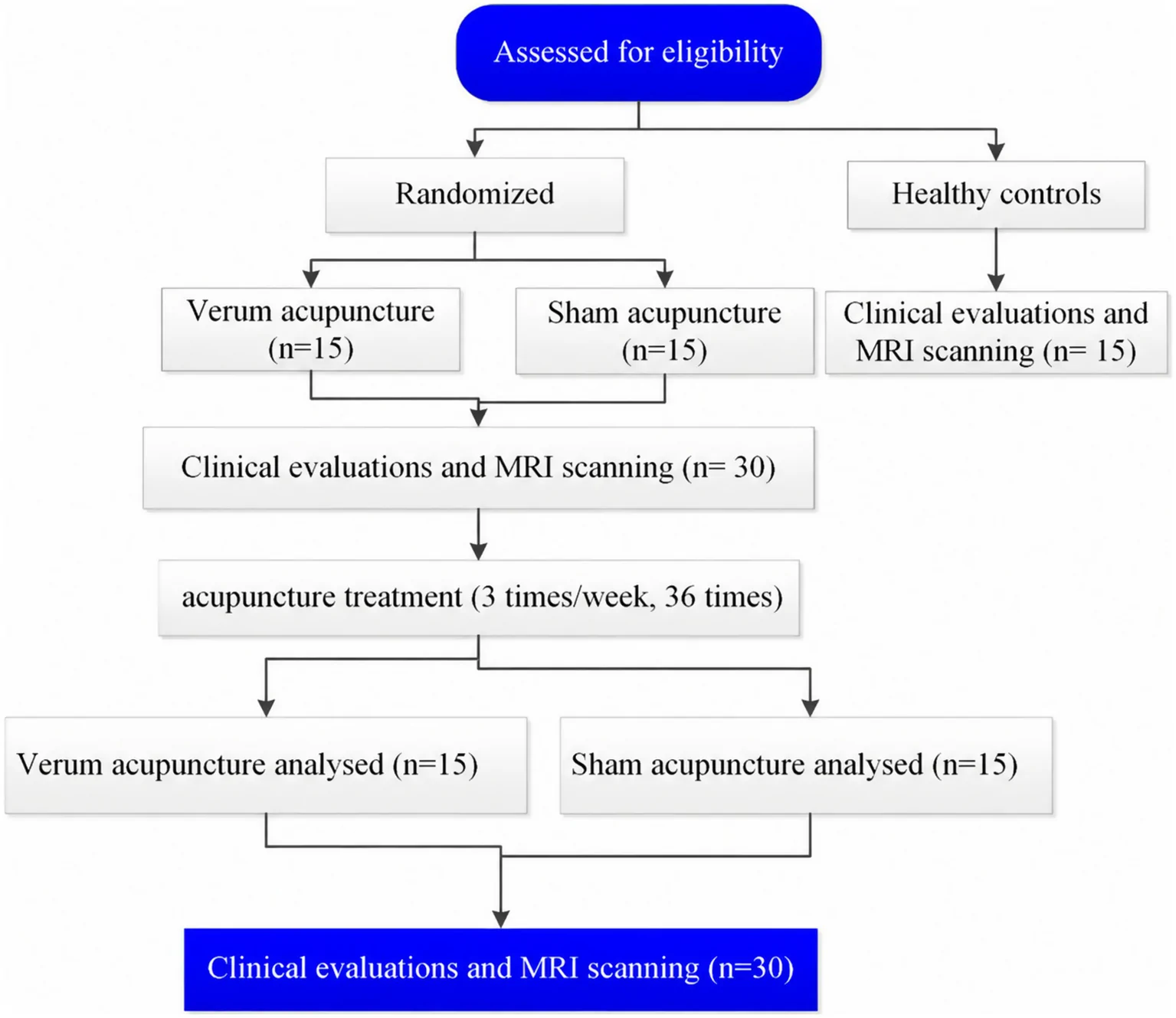
The flowchart.

**Table 1:** Demographics of participants.

Items	Healthy controls (*n* = 15)	Verum acupuncture (*n* = 15)	Sham acupuncture group (*n* = 15)	F/X^2^value	*p* value
Age (mean ± SD)	59.13 ± 6.49	61.93 ± 6.41	60.20 ± 5.89	1.984	0.150
Sex (male/female)	1/14	3/12	4/11	2.128	0.345
Education	12.93 ± 2.91	12.87 ± 2.67	11.40 ± 3.42	1.238	0.300

^a^One-Way ANOVA analysis (F value) was used for age of the three group, ^b^Chi-square analysis (X2 value) was used for sex and education of the three groups.

Following treatment, both the VA and SA groups showed increases in MMSE, MoCA, and AVLT scores and decreases in PSQI scores. Two-way repeated-measures ANOVA revealed a significant group × time interaction for PSQI scores (*F* = 76.14, *p* < 0.001), indicating a greater improvement in subjective sleep quality in the VA group than in the SA group. Significant group × time interactions were also observed for MMSE (*F* = 10.31, *p* = 0.003) and MoCA scores (*F* = 22.16, *p* < 0.001), demonstrating greater improvements in global cognitive function following verum acupuncture. HAMD scores also showed a significant group × time interaction (*F* = 4.48, *p* = 0.043), suggesting a greater reduction in depressive symptoms in the VA group.

Regarding memory performance, a significant group × time interaction was observed for the AVLT immediate recall composite score (*F* = 9.31, *p* = 0.005), indicating greater improvement in immediate verbal learning and memory following verum acupuncture. However, no significant group × time interactions were found for the individual AVLT subitems (AVLT-N1–N7; all *p* > 0.05) (Table 2).

**Table 2:** Clinical data results in aMCI patients.

Items	Verum acupuncture			Sham acupuncture			*P* value ^b^	Group × Time interaction
	Before VA	After VA	*P* value ^a^	Before SA	After SA	*P* value ^a^		
PSQI	12.33 ± 2.89	5.13 ± 1.30	<0.001	12.67 ± 2.19	11.93 ± 1.83	0.085	<0.001	*F* = 76.14, *p* < 0.001
MoCA	22.20 ± 2.31	25.93 ± 1.53	<0.000	22.80 ± 1.52	23.70 ± 1.53	0.512	<0.001	*F* = 10.31, *p* = 0.003
MMSE	26.07 ± 1.75	28.60 ± 1.68	<0.001	26.73 ± 1.53	27.27 ± 1.28	0.240	0.003	*F* = 22.16, *p* < 0.001
HAMD	7.27 ± 3.13	4.40 ± 2.26	0.001	4.67 ± 2.72	3.87 ± 3.29	0.276	0.043	*F* = 4.48, *p* = 0.043
AVLT-N1	4.33 ± 1.29	5.87 ± 1.68	0.003	3.13 ± 1.19	4.73 ± 2.09	0.015	0.926	*F* = 0.01, *p* = 0.93
AVLT-N2	5.87 ± 1.68	7.20 ± 1.86	0.045	5.40 ± 1.12	6.20 ± 1.70	0.075	0.475	*F* = 0.53, *p* = 0.48
AVLT-N3	6.80 ± 1.82	8.60 ± 1.76	0.002	6.20 ± 1.74	7.33 ± 1.68	0.039	0.343	*F* = 0.93, *p* = 0.34
Immediate recall	17.00 ± 4.16	21.67 ± 4.95	<0.001	14.73 ± 2.99	18.27 ± 4.98	<0.001	0.005	*F* = 9.31, *p* = 0.005
AVLT-N4	4.67 ± 1.84	7.47 ± 2.17	<0.001	4.40 ± 1.40	6.07 ± 1.83	0.008	0.125	*F* = 2.50, *p* = 0.13
AVLT-N5	3.33 ± 1.54	6.73 ± 2.49	<0.001	3.67 ± 1.05	5.60 ± 1.88	0.001	0.09	*F* = 3.08, *p* = 0.09
AVLT-N6	5.47 ± 2.47	8.07 ± 2.52	0.001	4.20 ± 1.66	5.73 ± 1.67	0.004	0.165	*F* = 2.04, *p* = 0.17
AVLT-N7	20.80 ± 2.24	21.87 ± 2.97	0.135	20.67 ± 2.06	21.40 ± 1.45	0.094	0.675	*F* = 0.18, *p* = 0.68

SD, standard deviation; MMSE, Mini-mental State Examination; MoCA, Montreal cognitive assessment; AVLT, auditory verbal learning test; a represents paired *t* tests; b represents two sample *t* tests.

### 3.2 Brain function indicators

Compared with the HC group, patients with aMCI and sleep disorders exhibited significantly reduced ALFF values in the left inferior temporal gyrus, right angular gyrus, and right dorsolateral superior frontal gyrus, as well as significantly increased ALFF values in the right caudate nucleus, left precuneus, and left anterior cingulate cortex. In addition, ReHo values were significantly decreased in the right cuneus and increased in the left cerebellum and right caudate nucleus (Table 3; Figures 2, 3).

**Table 3:** Comparison of brain function index differences among three groups of subjects.

Indicator	Group	AAL brain region	Peak MNI coordinate			Peak *t*-value	Cluster size (voxels)
			X	Y	Z		
ALFF	aMCI<HC	Right caudate nucleus	6	18	0	−5.06121	72
		Left precuneus	−3	−57	66	−4.72393	214
		Left anterior cingulate cortex	−6	27	18	−4.00462	65
	aMCI>HC	Left inferior temporal gyrus	−54	−18	−33	3.18310	79
		Right angular gyrus	60	−60	33	3.71705	71
		Right dorsolateral superior frontal gyrus	21	63	21	3.31169	154
ReHo	aMCI<HC	Left cerebellum	−3	−60	−48	−4.92441	53
		Right caudate nucleus	18	24	3	−4.69802	100
	aMCI>HC	Right cuneus	15	−78	33	4.02303	120

AAL, anatomical automatic labeling; MNI, montreal neurological institute; ALFF, amplitude of low-frequency fluctuation; ReHo, regional homogeneity; aMCI, amnestic mild cognitive impairment; HC, healthy controls.

**Figure 2:**
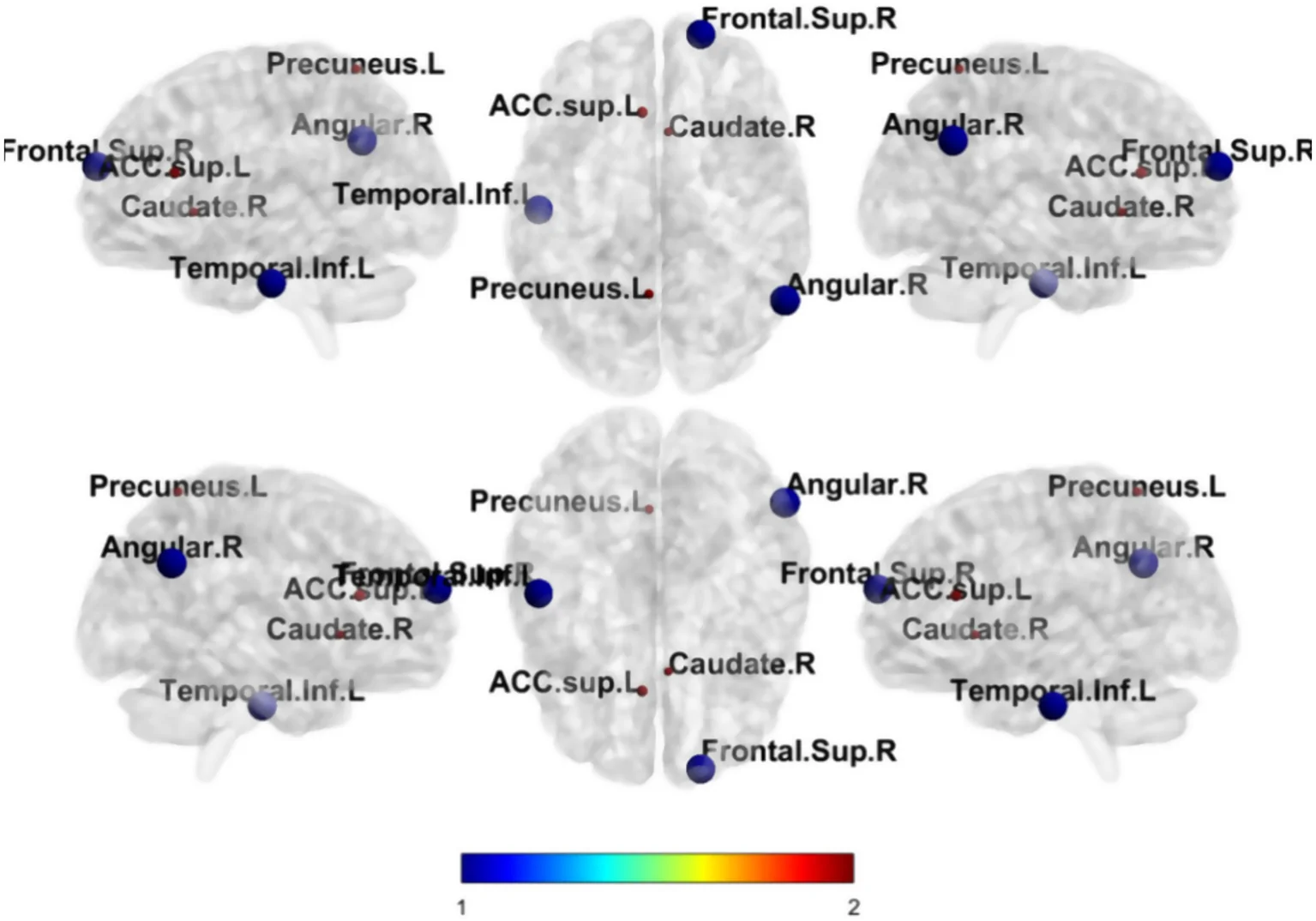
Brain regions showing ALFF differences between healthy controls and aMCI patients. Temporal.Inf.L, Left Inferior Temporal Gyrus; Caudate.R, Right Caudate Nucleus; Precuneus.L, Left Precuneus; Angular.R, Right Angular Gyrus; Frontal.Sup.R, Right Dorsolateral Superior Frontal Gyrus; ACC.sup.L, Left Anterior Cingulate Cortex; Blue indicates decreased ALFF, Red indicates increased ALFF.

**Figure 3:**
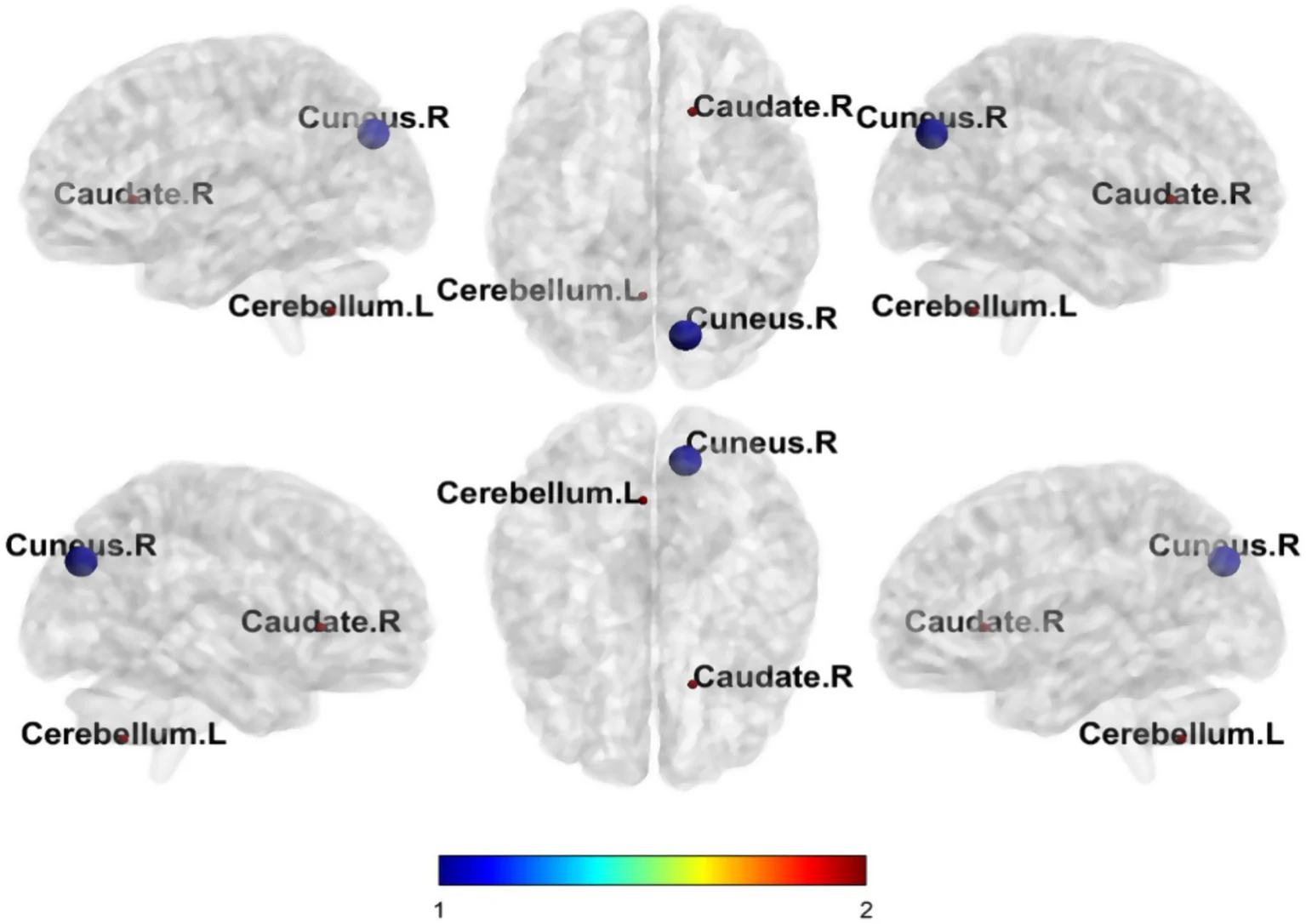
Brain regions showing ReHo differences between healthy controls and aMCI patients. Cuneus.R, Right Cuneus; Caudate.R, Right Caudate Nucleus; Cerebellum.L, Left Cerebellum; Blue indicates decreased ReHo, Red indicates increased ReHo.

Analysis of the group × time interaction revealed that VA significantly increased ALFF values in the left calcarine sulcus, whereas SA showed an opposite trend. Similarly, VA significantly increased ReHo values in cerebellar vermis lobule VI, while SA resulted in a decrease in this region. The between-group comparison further demonstrated that both groups showed reduced ALFF values in the right inferior temporal gyrus after treatment, although no significant group difference was observed in this region (Table 4).

**Table 4:** Bivariate repeated measures analysis results of ALFF and ReHo before and after acupuncture treatment.

Indicator	Group	AAL brain region	Peak MNI coordinate			Peak *t*-value	Cluster size (voxels)
			X	Y	Z		
ALFF	Treatment*Time interaction (Cluster 1)	Left Calcarine Sulcus	0	−99	3	−8.15965	97
	Treatment*Time between-group effect (Cluster 1)	Right inferior temporal gyrus	54	−45	−15	12.2959	114
ReHo	Treatment*Time interaction (Cluster 1)	Cerebellar Vermis VI	0	−78	−15	−5.3477	27

AAL, anatomical automatic labeling; MNI, montreal neurological institute; ALFF, amplitude of low-frequency fluctuation; ReHo, regional homogeneitya.

To further characterize treatment-related alterations, regions showing significant group differences were selected as ROI. After controlling for age, sex, and years of education, ROI values were extracted and compared before and after treatment within the VA. Paired *t*-test analyses revealed a significant increase in ALFF values within the left calcarine sulcus following VA treatment (*p* < 0.001). In addition, ReHo values in cerebellar vermis lobule VI were significantly elevated after VA treatment compared with baseline (*p* = 0.001) (Figure 4).

**Figure 4:**
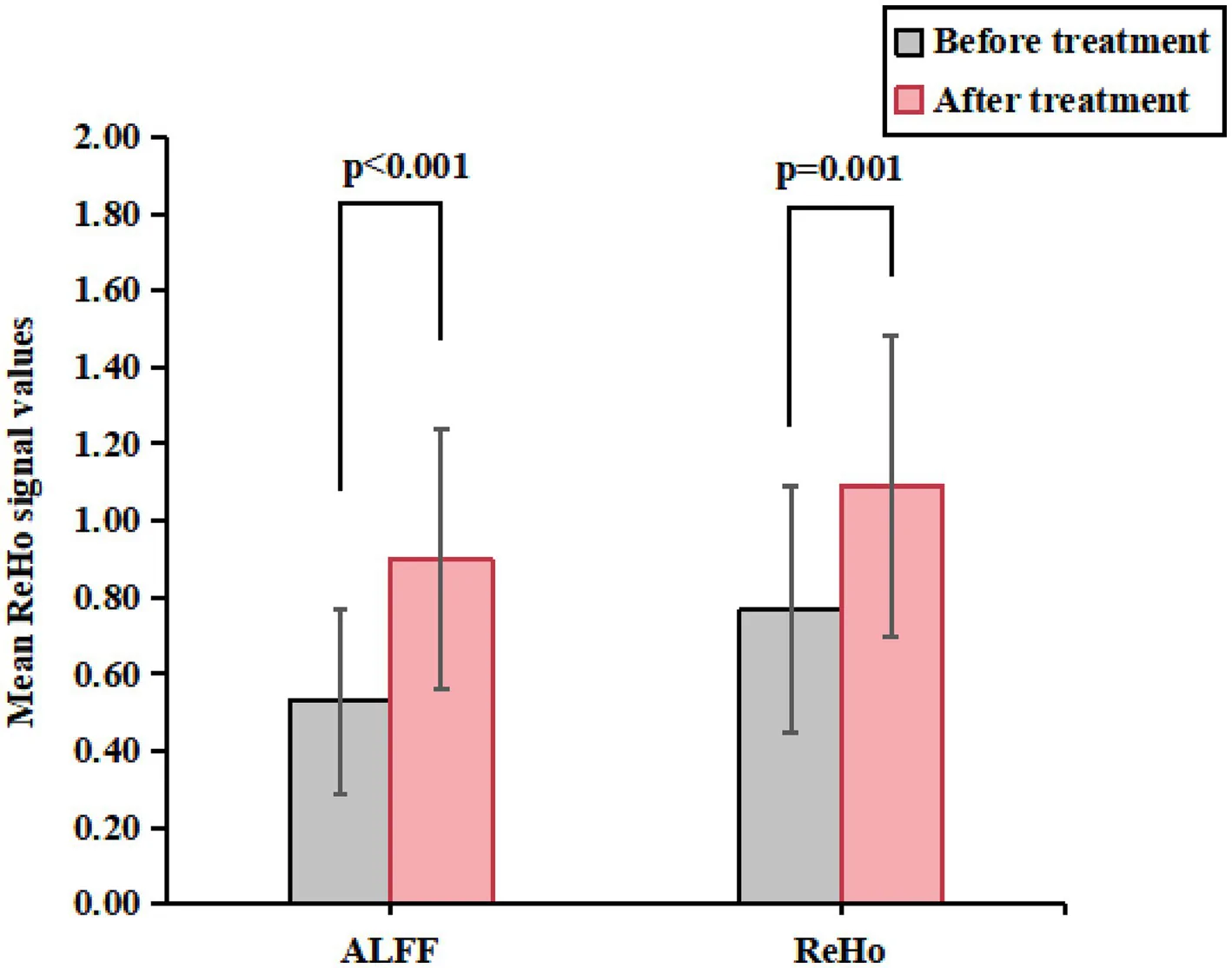
Changes in ROI values of brain regions before and after treatment in the VA group. ALFF difference brain regions before treatment (0.53 ± 0.24), after treatment (0.90 ± 0.32), *t*-value 5.322; compared with the ROI values before and after treatment, *p* < 0.001. ReHo difference brain regions before treatment (0.77 ± 0.34), after treatment (1.09 ± 0.39), *t*-value 4.105; compared with the ROI values before and after treatment, *p* = 0.001. ROI, region-of-interest; ALFF, amplitude of low-frequency fluctuation; ReHo, regional homogeneity. VA, verum acupuncture.

### 3.3 Correlation analysis

In the VA group, longitudinal changes were calculated as post-treatment values minus pre-treatment values. Increases in ALFF in the left calcarine sulcus were positively correlated with improvements in MoCA scores (*r* = 0.721, *p* = 0.003), but not with changes in PSQI scores (*r* = −0.461, *p* = 0.083). Similarly, increases in ReHo in cerebellar vermis VI were positively correlated with improvements in MoCA scores (*r* = 0.608, *p* = 0.013), whereas no significant correlation was found with changes in PSQI scores (*r* = −0.392, *p* = 0.148). In addition, changes in PSQI scores were negatively correlated with changes in MoCA scores (*r* = −0.536, *p* = 0.039), indicating that greater reductions in PSQI scores were associated with greater improvements in cognitive performance (Figure 5).

**Figure 5:**
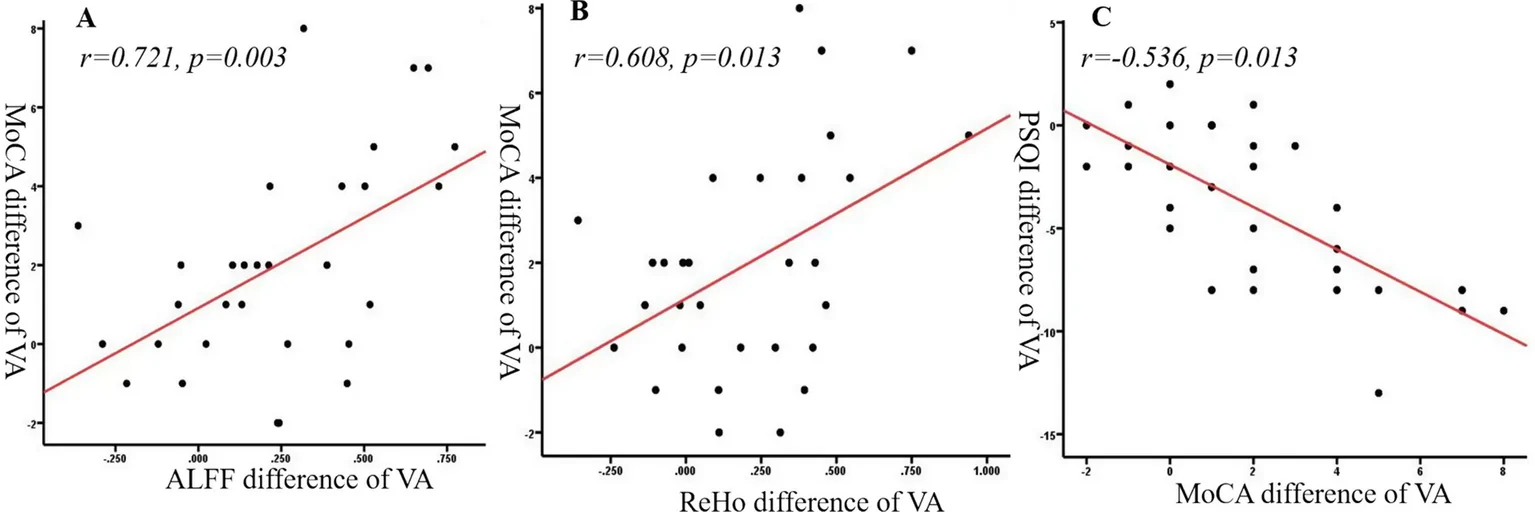
Correlation results. **(A)** MoCA difference was positively correlated with ALFF difference in VA group. **(B)** MoCA difference was positively correlated with ReHo difference in VA group. **(C)** MoCA difference was positively correlated with ALFF difference in VA group. **(B)** MoCA difference was negatively correlated with PSQI difference in VA group.

## 4 Discussion

### 4.1 Brain functional changes in aMCI patients with sleep disorders

This study revealed that, compared with healthy controls, aMCI patients with sleep disorders exhibited abnormal ALFF and ReHo in multiple brain regions. Specifically, these patients showed decreased functional activity in the inferior temporal gyrus, angular gyrus, cuneus, and dorsolateral superior frontal gyrus, along with increased activity in the caudate nucleus, precuneus, and cerebellum. These functional alterations are closely associated with the pathophysiology of cognitive-sleep disorders.

The inferior temporal gyrus may be involved in visual information processing and memory consolidation; its activity increases during rapid eye movement (REM) sleep (30), whereas activity in the inferior temporal gyrus is reduced in patients with mild cognitive impairment (aMCI) compared to healthy individuals. This may be due to a shortened duration of REM sleep, which in turn affects memory (31).

The caudate nucleus plays an important role in the regulation of the sleep–wake cycle through dopaminergic neurotransmission, and its hyperactivity has been implicated in the hyperarousal state characteristic of insomnia (32). In addition to sleep regulation, the caudate is involved in procedural learning, executive functioning, and attentional control (33). Altered caudate functional connectivity has been reported in patients with aMCI and is associated with deficits in attention and cognitive performance (34, 35). In patients with sleep disorders, functional connectivity among the precuneus, cingulate cortex, and prefrontal regions is disrupted, which may contribute to deficits in working memory and cognitive processing (36). The angular gyrus, a key Default Mode Network (DMN), is involved in sleep spindle generation and sleep-dependent declarative memory consolidation; abnormalities in sleep spindle activity may impair memory encoding and consolidation during sleep (37). Furthermore, poor sleep quality has been associated with increased resting-state functional connectivity between the anterior cingulate cortex and anterior DMN regions, potentially reflecting maladaptive neural activity related to hyperarousal and emotional dysregulation in insomnia (38, 39). Sleep deprivation also reduces metabolic activity in the dorsolateral superior frontal gyrus, a region critical for executive control, working memory, and decision-making. Structural and functional alterations in this region have been linked to an increased risk of progression from aMCI to AD (40, 41). The cuneus is a hub for large-scale cognitive networks; relative hypometabolism in sleep disorders can lead to cognitive decline (42). Collectively, these findings suggest that dysfunction of sleep–cognition shared brain networks may represent a key neurobiological mechanism underlying the coexistence of insomnia and cognitive impairment.

### 4.2 Neural mechanisms associated with acupuncture-induced improvements in cognition and sleep disorders

Acupuncture has demonstrated beneficial effects on both sleep disturbances and cognitive impairment, with accumulating evidence supporting its clinical efficacy. Previous studies suggest that acupuncture may modulate autonomic nervous system activity, reduce hypothalamic–pituitary–adrenal (HPA) axis hyperactivation, and influence cortical excitability and large-scale brain network organization, thereby contributing to improvements in sleep quality and cognitive performance (43, 44). In the present study, improvements in both clinical outcomes and neuroimaging indices were significantly greater in the verum acupuncture group than in the sham acupuncture group, supporting the specific therapeutic effects of acupuncture beyond nonspecific placebo-related factors.

Our findings further suggest that improvements in sleep may contribute to cognitive enhancement. Sleep disturbances are increasingly recognized as both a risk factor for and a consequence of cognitive decline (22). Notably, the calcarine cortex, a key component of the visual network (VN), has been implicated in attentional processing and cognitive performance (45). We observed that ReHo values in the left calcarine cortex were positively correlated with MoCA scores, while treatment-related changes in this region were associated with PSQI improvement. These findings indicate that modulation of visual network activity may represent a potential neural mechanism through which acupuncture simultaneously improves sleep quality and cognitive function.

In addition, increasing evidence suggests that the cerebellum contributes not only to motor control but also to higher-order cognitive and emotional processes through extensive cerebello-cortical connections (46). The cerebellar vermis, in particular, has been linked to executive function, emotional regulation, and sleep-related neural activity (47, 48). Experimental studies further suggest that the cerebellum interacts with limbic and brainstem structures involved in sleep regulation, including the amygdala, thalamus, and vestibular nuclei, and may participate in REM sleep-related neural processing (49). In the present study, increased ReHo values in the cerebellar vermis following acupuncture were significantly associated with improvements in sleep-related outcomes. These findings suggest that acupuncture-induced functional alterations in the cerebellar vermis may reflect modulation of the cerebellum–cortical network, potentially enhancing neural information integration and contributing to the improvement of both sleep and cognitive function.

Taken together, these findings support the hypothesis that acupuncture exerts therapeutic effects through the modulation of sleep–cognition shared brain networks. By regulating functional activity within visual, cerebellar, and cognitive control systems, acupuncture may facilitate the restoration of neural network homeostasis, ultimately leading to concurrent improvements in sleep quality and cognitive performance in patients with aMCI and sleep disturbances.

### 4.3 Limitations

Several limitations should be acknowledged. First, the relatively small sample size may have limited statistical power and generalizability, and larger multicenter studies are needed to validate these findings. Second, only short-term effects of acupuncture were evaluated, and the absence of long-term follow-up prevents assessment of treatment durability. Third, although the Streitberger placebo needle was used to maintain participant blinding, the 12-week treatment period and the absence of deqi in the sham acupuncture group may have enabled some participants to infer their treatment allocation. Because the success of blinding was not formally assessed, potential blinding failure and its influence on subjective outcomes cannot be completely excluded. Moreover, the relatively limited spatial resolution of fMRI, particularly the anisotropic voxel size (3.75 × 3.75 × 4.0 mm^3^), may have introduced partial volume effects and constrained the accurate localization of small regions such as the cerebellar vermis VI and caudate nucleus. Future studies using higher-resolution imaging are warranted. Finally, the neural mechanisms underlying acupuncture-induced modulation of sleep–cognition networks remain unclear. Integrating multimodal imaging with molecular, genetic, and multi-omics approaches may further elucidate these mechanisms.

## 5 Conclusion

In conclusion, acupuncture effectively improves sleep and cognitive function in aMCI patients. The therapeutic mechanism may be associated with alterations in functional activity within brain regions such as the left parietal sulcus and the cerebellar vermis, suggesting that the visual pathway and the cerebellum-cortex circuit may play a role in acupuncture’s ability to improve sleep quality and cognitive function. The correlation between cognitive and sleep improvements further supports the clinical relevance of combined interventions.

## Data Availability

The raw data supporting the conclusions of this article will be made available by the authors, without undue reservation.
