# Supplementary material for: Efficacy of acupuncture for sleep disorders in amnestic mild cognitive impairment: a randomized controlled trial with fMRI
Source: Front Neurol. 2026 Oct 6;17:1913478. doi: 10.3389/fneur.2026.1913478 (PMC13680174; doi:10.3389/fneur.2026.1913478)
Supplement: Supplementary file 1 [file Table_1.DOCX]

GdFigure\]

Figure S1. Brain regions showing ALFF differences between healthy controls and aMCI patients.

Figure S2. Brain regions showing ReHo differences between healthy controls and aMCI patients.

**Table S1.** Comparison of brain functional indices between HC and aMCI patients with sleep disorders

| Indicator | AAL brain region | Peak MNI  coordinate | | | Peak  t-value | Cluster size  (voxels) |
| --- | --- | --- | --- | --- | --- | --- |
|  |  | X | Y | Z |  |  |
| ALFF | Left Inferior Temporal Gyrus | -54 | -18 | -33 | 3.18310 | 79 |
|  | Right Caudate | 6 | 18 | 0 | -5.06121 | 72 |
|  | Left Precuneus | -3 | -57 | 66 | -4.72393 | 214 |
|  | Right Angular Gyrus | 60 | -60 | 33 | 3.71705 | 71 |
|  | Right dorsolateral superior frontal gyrus | 21 | 63 | 21 | 3.31169 | 154 |
|  | Left Anterior Cingulate Gyrus | -6 | 27 | 18 | -4.00462 | 65 |
| ReHo | Left Cerebellum | -3 | -60 | -48 | -4.92441 | 53 |
|  | Right Cuneus | 15 | -78 | 33 | 4.02303 | 120 |
|  | Right Caudate | 18 | 24 | 3 | -4.69802 | 100 |
